# The big five personality traits and eating habits among female students at Zayed University

**DOI:** 10.3389/fpubh.2024.1490634

**Published:** 2025-01-15

**Authors:** Sharifa AlBlooshi, Kawther AlSabbah, Shatha Thani, Rafiq Hijazi, Ayesha S. Al Dhaheri, Falak Zeb, Leila Cheikh Ismail

**Affiliations:** ^1^Department of Health Sciences, College of Natural and Health Sciences, Zayed University, Dubai, United Arab Emirates; ^2^Department of Mathematics and Statistics, College of Natural and Health Sciences, Zayed University, Abu Dhabi, United Arab Emirates; ^3^Department of Nutrition and Health, College of Medicine and Health Sciences, United Arab Emirates University, Al Ain, United Arab Emirates; ^4^Nutrition and Food Research Group, Research Institute of Medical and Health Sciences, University of Sharjah, Sharjah, United Arab Emirates; ^5^Department of Clinical Nutrition and Dietetics, College of Health Sciences, University of Sharjah, Sharjah, United Arab Emirates; ^6^Nuffield Department of Women’s and Reproductive Health, University of Oxford, Oxford, United Kingdom

**Keywords:** extraversion, conscientiousness, agreeableness, openness, neuroticism, personality traits, eating habits, UAE students

## Abstract

**Background:**

Adhering to healthy dietary habits is crucial for disease prevention and improving overall quality of life. Understanding how personality traits influence eating behaviors is essential for developing effective interventions aimed at promoting healthier eating habits. Personality traits are consistent behavioral patterns that individuals typically exhibit, and the Five-Factor Model (also known as the Big Five) is widely recognized as an effective framework for predicting personality traits.

**Methods:**

This study analyzed the relationship between personality traits and eating behaviors among 425 female students using a cross-sectional design. Sociodemographic data and personality traits were assessed using an online questionnaire, while eating behaviors were evaluated with the validated Three-Factor Eating Questionnaire (TFEQ). Key aspects of eating behavior studied included cognitive restraint, disinhibition, and emotional eating. The findings provide insights into how personality characteristics may influence eating habits, offering potential implications for interventions targeting healthier eating behaviors.

**Results:**

The majority of participants scored highest on the personality trait of openness to experience. The study found that certain personality traits, particularly neuroticism and agreeableness, were linked to dietary habits. Specifically, personality traits were associated with emotional eating (*p* < 0.003), but not with cognitive restraint (*p* = 0.25) or disinhibition (*p* = 0.308). Participants with higher levels of agreeableness demonstrated significantly higher cognitive restraint (*p* = 0.041).

**Conclusion:**

Personality traits can influence eating habits, particularly emotional eating. However, further research is needed to identify individuals at risk for diet-related diseases and to determine the most effective intervention strategies. This study is the first of its kind conducted in the United Arab Emirates, contributing valuable insights into the relationship between personality traits and eating behaviors.

## 1 Introduction

Dietary habits are a person’s choices or judgments about the meals they eat. These include decisions on when, how much, what, and where to eat, which could be altered by social and cultural factors; they can also be defined as “conscious, collective, and repetitive behaviors, which lead people to select, consume, and use certain foods or diets” (1). College students are well known for their inadequate nutritional intake and diet quality. This trend of inappropriate dietary habits has been linked to several causes, including residency changes, time management or convenience, dining out, financial restrictions, familial norms, an obsession with controlling weight, and misconceptions about nutrition (2, 3). People need help to switch from unhealthy to healthy eating habits, as there is commonly a gap between nutritional awareness and actual dietary intake (4). Since eating habits are formed early in childhood, adopting preventative measures to live a healthy lifestyle is essential. Personality traits are distinctive patterns of conduct or behavioral features that are consistently displayed by an individual (5). One commonly held theory of personality suggests that five primary domains or dimensions of traits interact to generate personality and modify an individual’s response to the social environment (6, 7).

According to many modern personality psychologists, five primary domains or dimensions of traits could interact to generate personality and alter the social environment (6). These personality traits are frequently referred to as the Big Five or the “Five Factor Model” of characteristics (8). The “Five Factor Model” has been shown to consistently predict personality qualities across interviews, self-descriptions, and physical observations, accounting for many personality traits without overlapping with other traits (9). The five categories include extraversion, agreeableness, conscientiousness, neuroticism, and openness to experience (9, 10). It may still be essential to consider how psychological factors like emotions or personality traits could modify eating habits. Eating behaviors frequently reflect a person’s mood and conduct. In an argument, Nicholls might suggest that there is a connection between emotions and eating patterns, claiming that just as eating can control emotions, emotions can modify eating (11).

It has been suggested that one’s personality traits are linked with eating behaviors (12–14). Previous research has shown a link between personality, specifically the Big Five Personality Traits, and nutritional behaviors, including eating habits and nutritional status (15–18). For instance, neuroticism was found to be negatively linked with healthy eating and habits (19). Moreover, a connection between eating disorders, body weight, and personality has been found in some studies. In one of these studies, food disinhibition was discovered to be closely related to adult weight increase (20). In addition, according to Strahler and colleagues, various psychological aspects have been linked to personality traits and some eating behaviors (21). Other studies also support the link between personality traits, gender, and eating habits. In one investigation, it was concluded that men had worse eating habits than women. Women tended to eat healthier diets than men, usually engaged in health-promoting behaviors, and led healthier lifestyles (22, 23). Additionally, in a study of 288 students, 16–18 years old, at Pertanian Bogor Institute, the multiple linear regression results revealed that the students who were well socialized (extroversion), creative and imaginative (openness to experience), and friendly and environmentally sensitive (agreeableness) tended to follow healthy eating habits and chose to eat vegetables (24). Moreover, another study revealed that extraversion was linked with restrained eating, agreeableness was linked with external and emotional eating, and openness to experience was associated with external, emotional, and restrained eating (25).

While multiple studies have shown how personality and eating habits are related, and numerous studies have found notable links between personality traits and outward appearance, particularly a person’s weight, the effect of different cultural factors remains unknown. The current study attempts to compare the findings on eating habits among university students in the United Arab Emirates with their personality traits in light of the diverse research noted above (20, 26). Additionally, particularly in the Arabian Gulf, little is known about how gender, personality traits, and eating habits interact. Therefore, studying the personality traits of college students may provide additional insight into what alters the quality of a diet (27, 28).

While prior research has consistently established a link between the Big Five personality traits and eating habits, much of this work has focused on populations in Western and non-Arab cultural contexts. Little is known about how personality traits interact with dietary behaviors in the Arabian Gulf region, particularly among young adults such as university students. Furthermore, limited research has examined how gender differences influence the relationship between personality traits and eating habits in culturally distinct settings like the United Arab Emirates (UAE). This study fills a critical gap by exploring the unique interplay between personality traits, cultural influences, and eating behaviors among students at Zayed University. By focusing on a population in the UAE, this research expands the understanding of how personality traits impact dietary habits in a non-Western, multicultural context, offering insights into the role of culture and gender in shaping these behaviors. These findings can contribute to culturally tailored interventions aimed at promoting healthier eating habits among university students.

This study aims to explore the relationship between personality traits and eating habits among students at Zayed University in the UAE, focusing on the Big Five Personality Traits and their influence on dietary behaviors. It examines the role of cultural influences unique to the UAE in shaping these behaviors. The study addresses gaps in understanding how psychological factors affect eating behaviors in non-Western contexts, the gendered dynamics of personality-related eating habits in the Arabian Gulf, and the interaction between psychological and sociocultural factors in dietary choices. It hypothesizes that certain traits, such as neuroticism and conscientiousness, significantly correlate with eating behaviors, and that cultural factors in the UAE affect these relationships.

## 2 Materials and methods

### 2.1 Ethical approval and clearance

The ethical standards of Zayed University were followed in this study. Ethical approval was obtained from the Research Ethics Committee at Zayed University, Code # ZU23_016_F. All participants were given an informed consent form to sign, which explained the purpose of the study. Confidentiality and anonymity were assured and guaranteed throughout the study, the participants were not subjected to any harm, and participation was voluntary.

### 2.2 Study design and area

The present cross-sectional study was conducted at Zayed University using an electronic survey. The sample included male and female students aged 18 and above. Students who were < 18 were excluded. The study was conducted over three months, from September to December 2023, in Dubai, United Arab Emirates.

### 2.3 Study participants and sampling

The study included undergraduate students, both local and international, from Zayed University who agreed to participate. Recruitment was conducted through convenience snowball sampling using an online questionnaire. Recruitment was conducted through convenience snowball sampling using an online questionnaire. A sample size of 456 students was chosen to ensure a maximum margin of error of 5% at a 95% confidence level, providing sufficient variability and statistical power to examine personality traits and eating habits in a diverse university population. While the inclusion of male and female, as well as local and international participants, initially aimed to enhance reliability, the 31 male respondents represented only about 7% of the sample, potentially introducing gender imbalance bias. Therefore, male participants were excluded, and the analysis was conducted exclusively on the 425 female respondents. This decision improved the internal validity of the study by ensuring a more homogenous and well-represented sample, though the non-random snowball sampling continues to limit generalizability. The sample size was also practical, aligning with similar studies and meeting resource and time constraints.

### 2.4 Data collection

An online survey was used in this study. A pilot study of 20 students was conducted to pre-test the questionnaire and ensure the validity of the questions before distributing it. The questionnaire consisted of two parts: the first was a test to determine personality type using the Big Five personality test. Researchers studying the fundamental characteristics of personality have often identified that variations in personality naturally fall into distinct categories. These traits have been shown to provide an accurate framework for understanding human nature. Most contemporary personality research is based on models that describe personality traits using scientifically validated dimensions. These models highlight distinct features in which individuals consistently vary. Since personality models represent latent variables, any trait can be further divided into smaller components, commonly referred to as facets.

The five traits—conscientiousness (orderly and achievement-oriented motivations), agreeableness (prosocial motivations to be empathic and to comply with norms), extraversion (enthusiasm and assertiveness), neuroticism (volatility and withdrawal), and openness (interest in ideas and aesthetics) (29–31). The respondents were asked to select the scale response that most accurately captured their attitude. The second part consisted of 48 questions divided into three sections. The first section included 8 questions about sociodemographic characteristics. The second section contained 20 behavioral and personal questions. The third section included 20 questions about cognitive practices, attitudes toward eating routines, and knowledge of the Big Five personality traits.

The study used validated tools to assess personality traits and eating behaviors. The Big Five Personality Traits Scale, adapted from the NEO-PI-R and aligned with cultural nuances, showed excellent reliability with Cronbach’s alpha values ranging from 0.80 to 0.90. The Three-Factor Eating Questionnaire (TFEQ) measured cognitive restraint, disinhibition, and emotional eating, with prior studies reporting Cronbach’s alpha values between 0.65 and 0.85 (7). The adaptation used for the study was based on a validated Arabic version developed to align with cultural nuances while maintaining the psychometric robustness of the original instrument.

### 2.5 Measurements

Data were obtained using a structured self-administered questionnaire. The data gathered included personality type, sociodemographic information, behavioral and personal information, cognitive practices, attitudes toward eating routines, and knowledge about the Big Five personality traits. Personality traits were assessed using a personality test questionnaire (7, 32, 33). Eating habits were evaluated using the “Three-Factor Eating Questionnaire” (TFEQ). The Three-Factor Eating Questionnaire is a validated questionnaire often applied in research on eating behavior, published by Stunkard and Messick in 1985 (43, 34, 35). The TFEQ measures 3 aspects of eating behavior: cognitive restraint of food intake, which involves tracking and controlling food intake and body weight; disinhibition of control of eating, which refers to the tendency to eat even after feeling full or satisfied; and emotional eating, which is eating as a coping mechanism for stress or in response to outside cues. The TFEQ comprised 18 items. Eight items measured cognitive restraint (CR), four measured disinhibition (uncontrolled eating) (UC), and the remaining three measured emotional eating (EE). Participants were required to select from 4-point Likert-type response scale (from 1 = “definitely false” to 4 = “definitely true”), the one that most accurately described them (36), except for item#18 that is scaled on a 8-point Likert-type response scale (1 = “no restraint in eating” to 8 = “total restraint”)— which will be recorded during the scoring procedure. Items scores are summed into three subscales: CR (6 items), UE (9 items), and EE (3 items). High scores on a scale reflect a higher level of that specific dimension. No total score should be computed (37).

### 2.6 Data analysis

Statistical data analysis was conducted with SPSS Version 29 (IBM Corporation, Armonk, NY, United States). Participants’ characteristics were summarized using frequencies and percentages. Multiple linear regression models were used to examine the associations between participants’ eating habits (disinhibition, cognitive restraint, and emotional eating) and their personality traits, while adjusting for sociodemographic factors, including age, nationality, marital status, and employment status. The significance of differences and relationships was established at a threshold of *p* < 0.05.

## 3 Results

### 3.1 Characteristics of participating students

Table 1 describes the characteristics of the students. A total of 425 female students participated. Most of the participants were UAE nationals (96.5%), unmarried (88.7%), had no chronic diseases (86.1%), and were aged between 18 and 25 (88.9%).

**Table 1:** The characteristics of respondents (*N* = 425).

Variables	Frequency	Percent (%)
Age		
18–25 years	378	88.9%
>25 years	47	11.1%
Nationality		
UAE national (Emirati)	410	96.5%
Non-UAE national (non-Emirati)	15	3.5%
Marital status		
Married	48	11.3%
Not married	377	88.7%
Employment status		
Employed	142	33.4%
Unemployed	283	66.6%
Chronic disease (having a chronic disease)		
Yes	59	13.9%
No	366	86.1%

Assessment of the strongest personality traits among the respondents reveals that openness to experience is the most prevalent, accounting for 33.4%. This is followed by conscientiousness (21.6%) and agreeableness (20.7%), which are nearly equally common. Extraversion accounts for 16.9%, while neuroticism is the least frequent trait, representing 7.3%.

### 3.2 Association between personality traits and dietary habits

Table 2 shows the multiple linear regression models of the dietary habits of the respondents, according to their personality traits, while controlling their demographic characteristics. The emotional eating model is significant at the 5% level, while the models for disinhibition and cognitive restraint are significant at the 10% level. Residual analysis for all three models indicated that the assumptions of homoscedasticity and normality were satisfied. Residual plots showed no clear patterns, and the Kolmogorov–Smirnov test revealed no substantial deviations from normality (*p* = 0.163, 0.153, and 0.024).

**Table 2:** Multiple linear regression models for dietary habits of respondents (*N* = 425).

	Disinhibition		Cognitive restraint		Emotional eating	
	Coefficient	*p*-value	Coefficient	*p*-value	Coefficient	*p*-value
Age (18–25 years)	0.027	0.800	0.005	0.954	0.010	0.919
Nationality (UAE national)	0.030	0.861	−0.059	0.677	0.097	0.558
Marital status (married)	−0.135	0.195	−0.046	0.590	0.007	0.942
Employment status (employed)	−0.170	0.014*	−0.145	0.011*	−0.106	0.110
Personality traits		0.308		0.250		<0.003*
Conscientiousness	0.112	0.199	0.044	0.544	0.278	0.001*
Extraversion	0.009	0.920	0.063	0.421	0.189	0.038*
Agreeableness	0.153	0.084	0.149	0.041*	0.283	<0.001*
Neuroticism	0.171	0.186	0.163	0.126	0.108	0.386
Openness to Experience	Ref		Ref		Ref	
Model utility test	*F*(8, 416) = 1.758, *p* = 0.084		*F*(8, 416) = 1.698, *p* = 0.097		*F*(8, 416) = 2.438, *p* = 0.014	
R-squared	R^2^ = 3.3%		R^2^ = 3.2%		R^2^ = 4.5%	

Overall, personality traits were a significant predictor of emotional eating (*p* < 0.003) but not of cognitive restraint (*p* = 0.25) and disinhibition (*p* = 0.308). With openness to experience as a reference group, participants with agreeableness tend to have significantly higher cognitive restraint scores (*p* = 0.041). On the other hand, participants with conscientiousness, extraversion, and agreeableness have significantly higher scores of emotional eating compared to their counterparts with openness to experience personality (*p* = 0.001, 0.038, and *p* < 0.001).

## 4 Discussion

This study reports on an area of nutrition research in the United Arab Emirates (UAE) that has not received much attention: the association between the Big Five personality traits of college students and their eating habits. The majority of students scored the highest on openness to experience, whose attributes include being imaginative and creative, open to unusual ideas, adventurous, and nonconformist. People with high scores in this dimension are self-reliant and prepared to accept a greater degree of uncertainty or ambiguity (38). Therefore, it is positive that many students displayed this quality. In this study, about 20.4% of participants scored high on conscientiousness. This result was opposite to that of Intiful et al., who found that a greater proportion of respondents scored higher on conscientiousness than on other traits (38). Individuals with high scores on conscientiousness can be described as self-controlled, responsible toward others, hard-working, organized, and rule-following (39). The cultural characteristics in the various studies may be the cause of the score variations. While Intiful et al.’s study focused on young adults in Ghana, this one primarily examined young adults in the United Arab Emirates. Because the United Arab Emirates is an open country and is distinguished by its political stability and diplomatic policy that is open to all countries of the world, its residents are characterized as being open as well (38).

In this study, agreeableness had higher scores on cognitive restraint and emotional eating. In another study, neuroticism had higher scores on non-recommended foods and a lower score on recommended foods (30). In addition, studies on the Big Five have shown that a number of personality traits, particularly neuroticism, which has been linked to the perception of one’s own body as larger than it actually is over time, have been important in explaining body dissatisfaction (24, 25, 40). Conscientiousness had a higher score in emotional eating, similar to other studies in which conscientiousness has been linked to healthy eating behaviors, such as regular eating time (41), where individuals with high conscientiousness scores were more receptive to dietary advice and adopting healthy practices and less engaged in counter-regulatory emotional or external eating (28, 42). In another study, conscientiousness had a negative association with body image dissatisfaction, similar to this study (25). Extraversion had a higher score in emotional eating, in contradiction to other studies in which it was correlated with restrained eating (30). Also, agreeableness could alter emotional eating like other similar studies where it was linked with emotional eating (30). Agreeableness is the general concern for social harmony, agreeable people value harmony with others. They are helpful, understanding, kind, giving, trustworthy, and prepared to put others’ interests ahead of their own. For this reason, when they feel betrayed by someone whom they have been kind to, they become emotional, which may lead to emotional eating. Apart from this, there is no current research describing the relationship between agreeableness and disinhibition. Although most participants of this study had openness to experience as their primary trait, there was no link between openness to experience and any of the eating habits. In contrast, Nelvi’s study found that emotional and restrained eating altered the openness to experience (30). It is difficult to explain why openness to experience was not substantially linked with any of the eating habits in our study.

### 4.1 Limitations

The responses to questions about dietary habits and personality traits among students might have been biased, as it was self-administered. Additionally, students’ stated eating habits might be impacted by other factors such as time constraints, the availability of food on campus, the demands of their academic work, and their purchasing power. Moreover, due to the lack of previous studies on this topic since there are few articles and studies done on the Big Five personality traits and eating habits, it was difficult to find a developed research methodology, and there was limited existing knowledge to build upon. This made it challenging to establish a strong theoretical framework or draw meaningful comparisons or conclusions.

Conducting the study as an online survey rather than face-to-face ensured a larger number of participants. However, this resulted in the researcher being unable to clarify any unclear questions, which may have led to inappropriate answers. There is evidence to support the relationship between personality traits and eating habits; however, further study is required to identify the people most at risk for diet-related illnesses so that individualized nutritional therapies based on their personality features can be developed. Individual’s conduct may be altered by their environment, the people they are in contact with, and their interactions, according to theories of personality and social psychology.

In the present study, eating habits were assessed using three dietary habits: emotional eating, cognitive restraint, and disinhibition. However, core food items such as sugary foods, salty foods, fatty foods, high-fiber foods, fruits, and vegetables were not assessed in the participants. Several studies have used the Food Frequency Questionnaire (FFQ) containing a larger number of food items to comprehensively investigate eating habits. One of the primary limitations of this study is the use of a snowball sampling strategy, a non-random procedure that does not ensure the representativeness of the sample. As a result, the findings cannot be generalized to the broader population with confidence. Future research should consider employing random sampling methods to enhance the representativeness and generalizability of the results.

Finally, since the current study only examined Zayed University students in the United Arab Emirates, no conclusions about the cultures or nations of other countries can be drawn. The results of previous studies and the current findings occasionally differed, suggesting that there may be culture-specific differences in the relationships between personality and eating habits.

## 5 Conclusion

To the best of our knowledge, this study is the first of its kind in the United Arab Emirates. Research has demonstrated the importance of personality in determining behavior. Investigating appropriate strategies to enhance dietary practices is crucial, given the rise in the incidence of chronic diseases caused by dietary changes.

The present investigation serves as an example of how the Big Five Personality Scale might be utilized to comprehend the eating habits of students in the UAE. Although it is restricted to a sample of Zayed University students, this study demonstrates that students with high neuroticism are more inclined toward cognitive restraint of food intake, which involves controlling food intake and tracking body weight. In comparison, students with high agreeableness are more inclined to emotional eating, which is eating as a coping mechanism for stress or in response to external cues, as well as disinhibition of control of eating, which refers to the tendency to eat even after feeling full or satisfied. Ultimately, it is advised that students understand the effects of both healthy and unhealthy eating behaviors.

The results of this study highlight the influence of personality traits on eating habits, providing a foundation for targeted interventions that address emotional and behavioral factors affecting dietary choices. These findings underscore the need for culturally tailored strategies in non-Western settings like the UAE, where unique sociocultural factors shape eating behaviors. However, the study’s limitations, including the use of snowball sampling and a specific university-based population, restrict the generalizability of the results. Future research should employ random sampling methods, expand the sample to include diverse populations, and explore underlying mechanisms, such as emotional and cognitive pathways, that mediate the relationship between personality traits and eating habits.

## Data Availability

The raw data supporting the conclusions of this article will be made available by the authors, without undue reservation.
